# Borderline liver enzyme patterns and their metabolic–inflammatory signatures: an observational outpatient study

**DOI:** 10.3389/fmed.2026.1838167

**Published:** 2026-05-15

**Authors:** Erdoğan Özdemir, Turgay Yılmaz

**Affiliations:** Department of Internal Medicine, Elazığ Fethi Sekin City Hospital, Elazığ, Türkiye

**Keywords:** borderline liver enzyme elevation, cholestatic pattern, hepatocellular pattern, MASLD, metabolic hyperferritinaemia, sex-specific ULN, systemic immune-inflammation index

## Abstract

**Background and aim:**

Liver enzyme elevations between one and two times the upper limit of normal (ULN) are common in outpatient practice and often dismissed as clinically insignificant, yet whether this range harbours meaningful subphenotypes, and whether the hepatocellular–cholestatic distinction applies at borderline levels, has not been systematically examined. We investigated whether, in adult outpatients with persistent borderline elevation confirmed using sex-specific ULN, the hepatocellular and cholestatic patterns carry distinct metabolic and inflammatory signatures.

**Materials and methods:**

This single-centre observational outpatient study was conducted at Elazığ Fethi Sekin City Hospital between October 2024 and January 2026. Eight hundred adults with standardised fasting biochemistry and haematology panels at baseline and third-month follow-up were analysed. Liver enzymes were classified as normal, borderline, or overtly elevated by the maximum fold-increase above the sex-specific ULN; borderline cases were subcategorised as hepatocellular, cholestatic, or overlap. Clinical, biochemical, inflammatory, and iron parameters were compared, and independent associations were examined using multivariable logistic regression.

**Results:**

Enzyme levels were normal in 548 patients (68.5%), borderline in 211 (26.4%), and overtly elevated in 41 (5.1%). Among the 211 borderline patients, 105 (49.8%) exhibited a hepatocellular pattern, 77 (36.5%) a cholestatic pattern, and 29 (13.7%) an overlap pattern. The hepatocellular pattern was associated with higher ferritin, serum iron, and transferrin saturation (TSAT), and a lower AST/ALT ratio; in multivariable analysis, ferritin was the only independent correlate (OR = 1.523; 95% CI: 1.199–1.935; *p* = 0.001). The cholestatic pattern showed higher HbA1c, higher systemic immune-inflammation index (SII), and lower TSAT; the independent predictors were HbA1c (OR = 1.591; 95% CI: 1.168–2.168; *p* = 0.003), SII (OR = 2.205; 95% CI: 1.228–3.960; *p* = 0.008), and TSAT (OR = 0.679; 95% CI: 0.489–0.943; *p* = 0.021), preserved in the non-diabetic subgroup. The overlap pattern exhibited features of both pure patterns.

**Conclusion:**

Persistent borderline liver enzyme elevation can be stratified into biochemically distinct subphenotypes when sex-specific reference ranges are applied. The cholestatic pattern is predominantly associated with glycaemic burden, systemic inflammation, and reduced iron transport, whereas the hepatocellular pattern shows an iron-predominant signature. A pattern-based approach may carry clinical value in outpatient management; findings from full models with limited events-per-variable, particularly for SII, are hypothesis-generating and require longitudinal validation.

## 1 Introduction

Elevated liver enzymes are among the laboratory findings most frequently encountered in outpatient practice (1). While marked elevations typically prompt a systematic evaluation, values between one and two times the upper limit of normal (ULN) are commonly dismissed as clinically insignificant and attributed to transient fluctuations, measurement error, or incidental findings of uncertain relevance (2). By contrast, the 2017 clinical guideline of the American College of Gastroenterology (ACG) defines this range as a “borderline” (<2 × ULN) category, highlights that it may be clinically relevant, and recommends reassessment within a few months (3).

The biological significance of this range has not been systematically addressed. The distinction between hepatocellular and cholestatic patterns has to date been examined primarily in the context of overt liver injury — particularly in drug-induced liver injury — and the two phenotypes have been shown to carry distinct clinical courses and outcomes (4). By contrast, large-scale clinical studies linking liver enzyme levels or their variability over time to cardiovascular events and mortality have treated enzyme elevation as a single category and have not incorporated the hepatocellular–cholestatic distinction into clinical risk stratification (5). The same approach persists in the setting of metabolic disease: enzyme elevation in MASLD patients has been shown to be strongly associated with long-term cardiometabolic mortality, yet this association has been evaluated independently of the underlying biochemical profile. Consequently, although the clinical relevance of this distinction is well defined in overt disease, it has not been specifically examined in the borderline range (6).

The biochemical basis for this distinction is important. The hepatocellular pattern is defined by AST and ALT; whereas ALT is relatively liver-specific, AST is also present in skeletal muscle, the heart, and erythrocytes, so its elevation is not always of hepatic origin (7). The cholestatic pattern is defined by ALP and GGT; ALP can also rise from extrahepatic sources such as bone, placenta, and intestine, while GGT, although more sensitive to the liver, is readily influenced by alcohol and enzyme-inducing medications (8). Despite these limitations, AST, ALT, ALP, and GGT remain the core markers used in the clinical evaluation of hepatocellular and cholestatic phenotypes, owing to their standardised reference ranges and inclusion in routine panels. Whether these two phenotypes carry distinct metabolic and inflammatory signatures at borderline levels is unknown.

Liver enzyme levels differ markedly by sex. The ACG 2017 clinical guideline defines the “healthy” ALT level as 29–33 IU/L in men and 19–25 IU/L in women, thereby formally endorsing the clinical use of sex-specific thresholds (3). This difference rests on a multifactorial biological foundation: the liver is one of the organs exhibiting the most pronounced sexual dimorphism, with sex hormones and growth hormone shaping liver-specific gene expression and metabolic activity, giving rise to distinct baseline enzyme profiles (9). This biological basis is also consistent with the sex-specific differences in prevalence and progression observed in metabolic liver diseases, most notably MASLD (formerly NAFLD) (10). Consequently, the use of a single threshold — particularly in women — carries the risk of systematically misclassifying enzyme elevations.

In light of these considerations, the present study aimed to examine, in adult outpatients with persistent borderline enzyme elevation confirmed by standardised repeat measurements and classified using sex-specific upper limits of normal, the metabolic and inflammatory signatures of the hepatocellular and cholestatic patterns in the outpatient setting.

## 2 Materials and methods

### 2.1 Study design and setting

This is a single-centre observational outpatient study based on standardised repeat measurements, conducted at the Department of Internal Medicine, Elazığ Fethi Sekin City Hospital, between October 2024 and January 2026. The study was approved by the Clinical Research Ethics Committee and carried out in accordance with the Declaration of Helsinki. Written informed consent was obtained from all participants. The study has been reported in accordance with the STROBE guideline for the reporting of observational studies.

#### 2.1.1 Inclusion criteria

Patients meeting all of the following criteria were included in the study:

- Age ≥18 years
- Adult outpatients presenting to the internal medicine clinic
- Availability of a complete, standardised fasting biochemistry and haematology panel at both the baseline and the three-month follow-up visit
- Provision of written informed consent

#### 2.1.2 Exclusion criteria

Patients with any of the following were excluded from the study:

- Missing key laboratory data
- Initiation or modification of any hepatotoxic medication within the 4 weeks preceding enrolment (including paracetamol, NSAIDs, antibiotics, antifungals, or statins)
- Oral contraceptive use (newly initiated or ongoing; to minimise hormonal confounding of enzyme reference ranges)
- Pre-existing chronic liver disease — viral hepatitis B or C, autoimmune hepatitis, primary biliary cholangitis, primary sclerosing cholangitis, alcohol-related liver disease, or cirrhosis of any aetiology — verified through ICD-10 diagnostic codes and the national patient tracking system
- Presence of symptoms suggestive of hepatobiliary disease at enrolment — including jaundice, right upper quadrant pain, pruritus, or unexplained fatigue — assessed by structured clinical history

The analysed cohort therefore comprised adult outpatients without clinical manifestations of hepatobiliary disease.

An *a priori* sample-size calculation was performed using G*Power 3.1.9.7 software (F tests—linear multiple regression: fixed model, R^2^ deviation from zero). Assuming a small-to-medium effect size (Cohen’s *f*^2^ = 0.10), a two-sided *α* of 0.05, and 90% power, the required minimum total sample sizes were 215 for the primary 10-predictor multivariable model, 199 for the pre-specified 8-predictor reduced pattern-specific model, and 267 for the pre-specified 18-predictor full pattern-specific model. These calculations were used to define the design requirements for the overall analytical framework, including the planned pattern-specific modelling strategy. Over the course of the study, 1,125 consecutive outpatients were screened. After applying the pre-specified inclusion and exclusion criteria, 325 patients were excluded, and the remaining 800 patients were included in the final analyses. A detailed breakdown of the screening process and reasons for exclusion is provided in Supplementary Figure S1 (STROBE-style flow diagram).

### 2.2 Classification of liver enzyme elevation

Sex-specific upper limits of normal (ULN) were defined for all four enzymes according to the standard reference ranges of our hospital laboratory: ALT (women >25 U/L, men >35 U/L), AST (women >25 U/L, men >35 U/L), GGT (women >35 U/L, men >55 U/L), and ALP (women >100 U/L, men >120 U/L). These thresholds are consistent with the ACG 2017 clinical guideline’s recommendation for the use of sex-specific ULN (3). Each patient was assigned to one of three groups based on the maximum fold-increase above the applicable sex-specific ULN across the four enzymes: normal (≤1 × ULN), borderline elevation (>1–<2 × ULN), and overt elevation (≥2 × ULN). Borderline cases were further divided into three groups according to pattern: hepatocellular (AST and/or ALT elevated, ALP and GGT normal), cholestatic (ALP and/or GGT elevated, AST and ALT normal), and overlap (both patterns present simultaneously). As an additional descriptor, the AST/ALT ratio was calculated.

### 2.3 Laboratory parameters and inflammatory indices

The routine baseline panel included the following parameters: complete blood count, liver enzymes (AST, ALT, ALP, GGT), creatine kinase, lactate dehydrogenase, amylase, albumin, renal function tests (urea, creatinine), glucose, HbA1c, lipid profile (total cholesterol, HDL, LDL, triglycerides), iron parameters (serum iron, ferritin, TSAT, UIBC), thyroid function tests (TSH, free T3, free T4), vitamin B12, 25-OH vitamin D, and CRP; all participants underwent abdominal ultrasonography. MASLD was defined according to the current criteria (11) as the coexistence of hepatic steatosis on imaging and at least one cardiometabolic risk factor. Within this framework, body mass index (≥25 kg/m^2^) was used in the anthropometric assessment, and a diagnosis of hypertension or use of antihypertensive medication was used in the blood pressure assessment. Biochemical analyses were performed using a Beckman Coulter AU5800 automated chemistry analyser, and complete blood count analyses were performed using a Beckman Coulter DxH 800 analyser. Intra-assay coefficients of variation were <2.5% for AST, ALT, and GGT, and <2.0% for ALP.

The following composite inflammatory indices were derived from routine haematological parameters: neutrophil-to-lymphocyte ratio (NLR), neutrophil-to-monocyte ratio (NMR), systemic immune-inflammation index (SII = platelet × neutrophil / lymphocyte), and aggregate index of systemic inflammation (AISI = neutrophil × monocyte × eosinophil / lymphocyte). FIB-4 and APRI were calculated as non-invasive fibrosis scores. eGFR was estimated using the CKD-EPI 2021 creatinine equation (12). Hepatic steatosis was graded by abdominal ultrasonography (absent, Grade 1, 2, or 3). Smoking was quantified in pack-years. Alcohol consumption was assessed by self-report and classified as harmful or non-harmful according to the EASL–EASD–EASO 2024 MASLD guideline (13). Comorbidity diagnoses and medication-use patterns were verified through patient self-report, ICD-10 diagnostic codes, the Central Physician Information System, and e-Prescription records. BMI was recorded as the mean of height/weight measurements obtained at the two visits.

### 2.4 Statistical analysis

Continuous variables are expressed as mean ± SD or median (IQR) according to distribution, which was assessed by the Shapiro–Wilk test. Two-group comparisons were performed using the Mann–Whitney U test or the independent-samples t-test, and multi-group comparisons were performed using the Kruskal–Wallis test. Categorical variables were compared using Pearson’s chi-square test or Fisher’s exact test. Associations between continuous variables were assessed by Spearman rank correlation. Binary logistic regression was applied to compare pattern subgroups with the normal group and with each other. A full multivariable model and a pre-specified reduced model were fitted in parallel. Continuous variables were standardised, and odds ratios (OR) are reported per 1-SD increment. Multicollinearity was evaluated using the variance inflation factor (VIF). Sex-by-covariate interaction terms were pre-specified and included in the models. As a pre-specified sensitivity analysis addressing potential collinearity between HbA1c and age, an age-residualisation procedure was additionally performed for the cholestatic model: HbA1c was first regressed on age using ordinary least squares (HbA1c = *β*₀ + β₁ × Age + *ε*), and the resulting residuals, representing the component of HbA1c variance orthogonal to age, were entered in place of raw HbA1c into the multivariable logistic regression model. Per the Frisch–Waugh–Lovell theorem, the partial coefficient of a covariate in a multivariable linear regression is mathematically equivalent to the coefficient obtained by first residualising that covariate against all other predictors; this analysis therefore provides an explicit test of whether the HbA1c–pattern association is attributable to shared variance with age. As a sensitivity analysis, the models were repeated in the subcohort without hepatic steatosis. Internal validity was assessed using non-parametric bootstrap with 1,000 iterations. For the cholestatic and hepatocellular subgroups, pattern-specific post-hoc statistical power for the independently significant associations was additionally estimated using Hsieh’s (1998) approximation for logistic regression, assuming standardised predictors and the observed event proportions. Given the limited events-per-variable in the overlap subgroup (*n* = 29; EPV < 2 with 18 covariates), an adjusted multivariable model was not fitted for this subgroup, which is accordingly reported descriptively. All tests were two-sided, and *p* < 0.05 was considered statistically significant. Analyses were performed using IBM SPSS Statistics version 26.0 (IBM Corp., Armonk, NY, United States).

## 3 Results

### 3.1 Cohort composition and pattern distribution

The cohort had a mean age of 50.4 ± 16.3 years, and 57.4% were female. Enzyme levels were normal in 548 patients (68.5%), borderline elevated (>1–<2 × ULN) in 211 (26.4%), and overtly elevated (≥2 × ULN) in 41 (5.1%) (baseline characteristics are presented in Supplementary Table S1). Within the borderline elevation group, 105 patients (49.8%) exhibited a hepatocellular pattern, 77 (36.5%) a cholestatic pattern, and 29 (13.7%) an overlap pattern. The baseline characteristics of the normal and borderline elevation groups are presented in Table 1.

**Table 1:** Baseline characteristics: normal versus borderline elevation groups.

Variable	Normal (*n* = 548)	Borderline (*n* = 211)	*p*
Age (years)	50.0 (38–63)	52.0 (41–63)	0.421
Female sex, *n* (%)	310 (56.6%)	130 (61.6%)	0.239
BMI (kg/m^2^)	28.4 (25.5–31.0)	29.0 (25.4–31.6)	0.183
HbA1c (%)	5.7 (5.3–6.3)	5.9 (5.5–7.2)	<0.001
TG/HDL ratio	2.6 (1.7–4.5)	3.1 (1.8–4.7)	0.091
Triglycerides (mg/dL)	131 (91–204)	163 (99–227)	0.004
Total cholesterol (mg/dL)	187 (159–214)	202 (170–230)	<0.001
Fasting glucose (mg/dL)	96 (86–115)	101 (89–149)	0.002
Ferritin (μg/L)	34.0 (15–64)	45.0 (20–90)	0.002
TSAT (%)	21.8 (15.3–29.8)	20.7 (13.5–29.1)	0.350
AISI	0.13 (0.07–0.26)	0.13 (0.07–0.26)	0.875
NLR	1.85 (1.48–2.38)	1.82 (1.48–2.42)	0.698
NMR	7.70 (6.24–9.28)	7.83 (6.31–9.51)	0.490
CRP (mg/L)	3.2 (1.8–5.4)	2.6 (1.6–5.8)	0.589
Albumin (g/L)	43.0 (41–45)	43.0 (41–46)	0.091
Haemoglobin (g/dL)	14.4 (13.4–15.5)	14.6 (13.5–15.8)	0.160
HCT (%)	42.1 (39.6–45.1)	42.8 (39.8–46.0)	0.095
eGFR (mL/min/1.73m^2^)	100.0 (89–113)	102.0 (90–115)	0.616
Hepatic steatosis, *n* (%)	321 (58.6%)	122 (57.8%)	0.915
Smoking, *n* (%)	188 (34.3%)	70 (33.2%)	0.834
Alcohol use, *n* (%)	63 (11.5%)	21 (10.0%)	0.632
Diabetes mellitus, *n* (%)	132 (24.1%)	72 (34.1%)	0.007

Mann–Whitney U test for continuous variables; Pearson chi-square or Fisher exact test for categorical variables. Continuous variables: median (IQR); categorical variables: n (%). The overt elevation group (*n* = 41, ≥2 × ULN) is excluded from this table and its baseline characteristics are provided in Supplementary Table S1. BMI, Body mass index; HbA1c, Glycated haemoglobin; TG/HDL, Triglyceride-to-HDL cholesterol ratio; HCT, Haematocrit; TSAT, Transferrin saturation; AISI, Aggregate index of systemic inflammation; NLR, Neutrophil-to-lymphocyte ratio; NMR, Neutrophil-to-monocyte ratio; CRP, C-reactive protein; eGFR, Estimated glomerular filtration rate.

### 3.2 Raw comparisons: pattern biochemical profiles

The proportion of participants meeting MASLD criteria was similar across the normal, hepatocellular, and cholestatic groups (55.8, 49.5, and 58.4%, respectively; 72.4% in the overlap group); no significant between-group difference was observed (chi-square *p* = 0.16).

Compared with the normal group, the hepatocellular pattern was characterised by higher levels of ferritin, serum iron, TSAT, creatine kinase, and LDH, together with a characteristically low AST/ALT ratio (all *p* < 0.05). Inflammatory indices (NMR, SII, NLR) were lower in the hepatocellular group than in normal controls (all *p* < 0.01).

In the cholestatic group, HbA1c, inflammatory indices (NMR, SII, NLR), white blood cell and neutrophil counts, total cholesterol, and fasting glucose were markedly higher, whereas serum iron and TSAT were lower (all *p* ≤ 0.018). The cholestatic pattern showed a female predominance (70.1% vs. 56.6% in the normal group, *p* = 0.033); no significant sex difference was observed in the hepatocellular pattern.

In the direct comparison between the two borderline patterns, the cholestatic pattern was characterised by higher HbA1c and inflammatory indices, whereas the hepatocellular pattern showed higher iron markers and creatine kinase together with a lower AST/ALT ratio (all *p* < 0.01). Patients in the cholestatic group were significantly older than those in the hepatocellular group (median 56 vs. 46 years, *p* < 0.001). All univariable comparisons are presented in Table 2.

**Table 2:** Raw comparison of biochemical profiles: hepatocellular and cholestatic patterns versus normal group and versus each other.

Variable	Normal (*n* = 548)	Hepatocellular (*n* = 105)	*p* ^†^	Cholestatic (*n* = 77)	*p* ^†^	*p* ^‡^
Ferritin (μg/L)	34.0 (15–64)	48.0 (21–100)	0.005	36.0 (17–80)	0.336	0.225
Serum iron (μg/dL)	76.0 (54–105)	85.0 (58–118)	0.025	67.0 (42–83)	0.002	<0.001
TSAT (%)	21.8 (15.3–29.8)	24.4 (16.3–33.9)	0.037	18.4 (10.5–23.3)	0.002	<0.001
CK (U/L)	82 (59–111)	106 (70–159)	<0.001	68 (52–87)	0.009	<0.001
LDH (U/L)	176 (155–202)	191 (171–216)	<0.001	184 (159–211)	0.111	0.133
AST/ALT ratio	1.17 (1.00–1.46)	0.77 (0.64–1.00)	<0.001	1.13 (0.91–1.31)	0.094	<0.001
Free T3 (pmol/L)	3.39 (3.1–3.7)	3.50 (3.2–3.8)	0.079	3.37 (3.1–3.7)	0.834	0.165
HbA1c (%)	5.70 (5.3–6.3)	5.70 (5.3–6.3)	0.672	6.10 (5.7–8.1)	<0.001	<0.001
Neutrophil (×10^9^/L)	4.05 (3.3–4.9)	3.80 (3.1–4.7)	0.036	4.75 (4.1–6.1)	<0.001	<0.001
WBC (×10^9^/L)	6.98 (5.9–8.2)	6.99 (5.5–8.2)	0.398	7.61 (6.5–9.3)	<0.001	<0.001
NMR	7.70 (6.2–9.3)	7.24 (5.4–8.5)	<0.001	8.92 (7.5–10.0)	<0.001	<0.001
SII	477 (350–642)	420 (312–539)	0.008	592 (459–781)	<0.001	<0.001
AISI	0.13 (0.07–0.26)	0.12 (0.07–0.22)	0.608	0.14 (0.06–0.30)	0.659	0.459
NLR	1.85 (1.5–2.4)	1.64 (1.3–2.1)	0.004	2.06 (1.6–2.8)	0.018	<0.001
CRP (mg/L)	3.2 (1.8–5.4)	2.6 (1.6–5.1)	0.133	3.1 (1.7–6.6)	0.732	0.176
Total chol. (mg/dL)	186 (159–214)	200 (166–230)	0.014	208 (180–230)	0.002	0.476
Fasting glucose (mg/dL)	96 (86–115)	96 (88–120)	0.719	106 (91–167)	<0.001	0.009
Albumin (g/L)	43 (41–45)	44 (42–46)	0.006	42 (40–45)	0.361	0.008
HCT (%)	42.1 (39.6–45.1)	43.1 (40.4–46.5)	0.035	41.9 (38.9–45.4)	0.790	0.112
Female sex, *n* (%)	310 (56.6%)	56 (53.3%)	0.614	54 (70.1%)	0.033	0.033
Age (years)	50.0 (38–63)	46 (38–58)	0.113	56 (47–66)	0.007	<0.001

^†^vs normal group; ^‡^hepatocellular vs cholestatic direct comparison. Mann–Whitney U for continuous variables; Pearson chi-square for categorical variables. Continuous variables: median (IQR). CK, Creatine kinase; LDH, Lactate dehydrogenase; HbA1c, Glycated haemoglobin; WBC, White blood cell count; TSAT, Transferrin saturation; NMR, Neutrophil-to-monocyte ratio; SII, Systemic immune-inflammation index; AISI, Aggregate index of systemic inflammation; NLR, Neutrophil-to-lymphocyte ratio; CRP, C-reactive protein; HCT, haematocrit.

### 3.3 Multivariable analysis: pattern predictors

In the multivariable logistic regression analysis adjusted for 18 variables, ferritin was identified as the only variable independently associated with the hepatocellular pattern (OR = 1.523 for standardised ferritin; 95% CI: 1.199–1.935; *p* = 0.001). In this model, no metabolic or inflammatory index reached independent significance (*n* = 653, EPV = 5.8, all VIF < 3.5).

**Figure 1:**
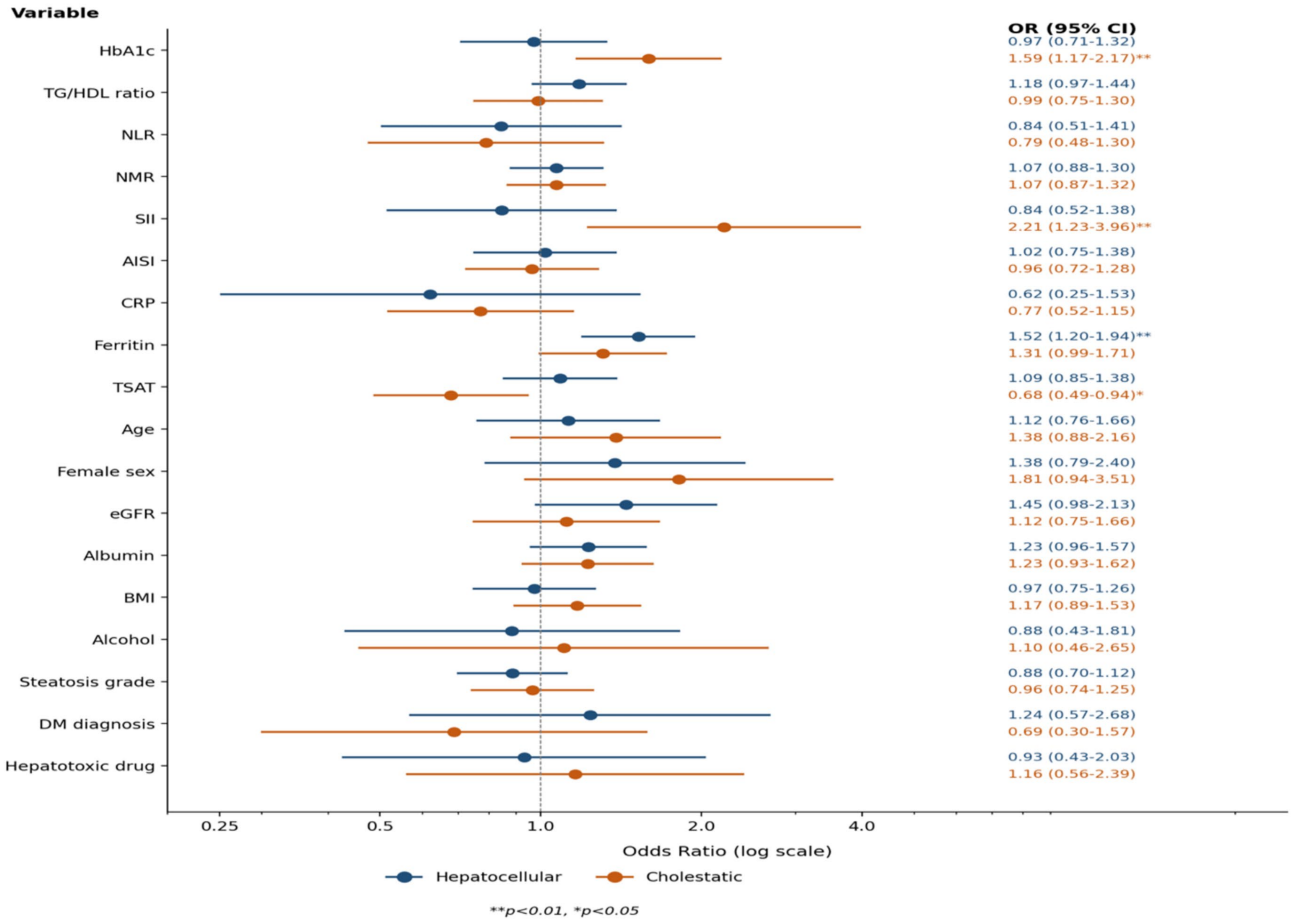
Forest plot of multivariable odds ratios for borderline hepatocellular and cholestatic patterns versus the normal group.

For the cholestatic pattern, HbA1c (OR = 1.591; 95% CI: 1.168–2.168; *p* = 0.003), SII (OR = 2.205; 95% CI: 1.228–3.960; *p* = 0.008), and TSAT (OR = 0.679; 95% CI: 0.489–0.943; *p* = 0.021) were identified as independent predictors (*n* = 625, EPV = 4.3). Owing to the limited EPV, a pre-specified eight-variable reduced model was applied (EPV = 9.6), in which HbA1c (OR = 1.456, *p* < 0.001), TSAT (OR = 0.614, *p* = 0.003), female sex (OR = 1.976, *p* = 0.026), and ferritin (OR = 1.345, *p* = 0.022) were confirmed. Directional consistency was preserved across both model specifications (Table 3). A forest-plot visualisation of the multivariable ORs is presented in Figure 1. A graphical summary integrating the pattern-specific signatures with their clinical interpretations is provided in Supplementary Figure S2.

**Table 3:** Multivariable logistic regression: hepatocellular and cholestatic patterns versus normal group.

Variable	Hepatocellular OR (95% CI)	*p*	Cholestatic OR (95%CI)	*p*	VIF
HbA1c	0.970 (0.711–1.323)	0.847	1.591 (1.168–2.168)	0.003**	2.2
TG/HDL ratio	1.180 (0.966–1.441)	0.105	0.989 (0.752–1.300)	0.936	1.1
NLR	0.843 (0.505–1.408)	0.514	0.789 (0.477–1.305)	0.356	3.5
NMR	1.070 (0.880–1.302)	0.496	1.069 (0.867–1.317)	0.534	1.0
SII	0.844 (0.517–1.378)	0.497	2.205 (1.228–3.960)	0.008**	2.4
AISI	1.017 (0.751–1.378)	0.911	0.962 (0.725–1.277)	0.791	1.3
CRP	0.620 (0.252–1.529)	0.299	0.771 (0.518–1.148)	0.201	1.5
Ferritin	1.523 (1.199–1.935)	0.001**	1.306 (0.995–1.713)	0.054	1.4
TSAT	1.086 (0.853–1.384)	0.502	0.679 (0.489–0.943)	0.021*	1.3
Age	1.125 (0.761–1.664)	0.554	1.381 (0.881–2.163)	0.159	2.9
Female sex	1.377 (0.790–2.403)	0.259	1.812 (0.935–3.511)	0.078	1.4
eGFR	1.445 (0.980–2.128)	0.063	1.116 (0.749–1.661)	0.590	2.5
Albumin	1.228 (0.959–1.572)	0.104	1.225 (0.926–1.620)	0.154	1.3
BMI	0.973 (0.750–1.262)	0.835	1.170 (0.893–1.533)	0.253	1.4
Alcohol	0.883 (0.431–1.811)	0.735	1.103 (0.458–2.653)	0.827	1.1
Steatosis grade	0.884 (0.700–1.116)	0.300	0.965 (0.744–1.251)	0.786	1.1
DM diagnosis	1.237 (0.571–2.678)	0.590	0.688 (0.301–1.573)	0.376	2.3
Hepatotoxic drug	0.931 (0.427–2.027)	0.857	1.159 (0.562–2.390)	0.690	1.2

Hepatocellular model: n = 653, events = 105, EPV = 5.8. Cholestatic model: *n* = 625, events = 77, EPV = 4.3. Reference category: normal group. All models adjusted for all listed variables. Continuous variables standardised; ORs expressed per 1 SD increment. Reduced cholestatic model (EPV = 9.6, 8 pre-specified variables): HbA1c OR = 1.456 (*p* < 0.001), TSAT OR = 0.614 (*p* = 0.003), female sex OR = 1.976 (*p* = 0.026), ferritin OR = 1.345 (*p* = 0.022) — directionally consistent with full model. OR: odds ratio; CI: confidence interval; VIF: variance inflation factor; HbA1c: glycated haemoglobin; TG/HDL: triglyceride-to-HDL cholesterol ratio; NLR: neutrophil-to-lymphocyte ratio; NMR: neutrophil-to-monocyte ratio; SII: systemic immune-inflammation index; AISI: aggregate index of systemic inflammation; CRP: C-reactive protein; TSAT: transferrin saturation; BMI: body mass index; eGFR: estimated glomerular filtration rate; DM: diabetes mellitus. *p < 0.05; *p < 0.01.

Adjusted odds ratios (OR) with 95% confidence intervals from separate binary logistic regression models comparing the borderline hepatocellular pattern (blue; *n* = 105) and the borderline cholestatic pattern (orange; *n* = 77) to the normal group (*n* = 548). Models were adjusted for 18 covariates (HbA1c, TSAT, ferritin, SII, NLR, NMR, AISI, CRP, albumin, age, sex, BMI, eGFR, TG/HDL ratio, alcohol use, hepatic steatosis grade, diabetes mellitus, and recent hepatotoxic medication use); continuous variables were standardised and odds ratios are expressed per 1 standard deviation increment. All 18 covariates entered in the full multivariable models are displayed; the corresponding numerical estimates are also provided in Table 3. The dashed vertical line indicates the reference value (OR = 1.0). OR: odds ratio; CI: confidence interval; HbA1c: glycated haemoglobin; SII: systemic immune-inflammation index; TSAT: transferrin saturation; eGFR: estimated glomerular filtration rate; TG/HDL: triglyceride-to-HDL cholesterol ratio. **p < 0.01, *p < 0.05.

Among non-diabetic participants, HbA1c was also significantly higher in the cholestatic group than in the normal group (median 5.70% vs. 5.50%, *p* = 0.001). In the pre-specified sensitivity analysis performed in the subcohort without hepatic steatosis (*n* = 333), ferritin remained independently associated with the hepatocellular pattern (OR = 2.57, *p* < 0.001); for the cholestatic pattern, HbA1c (OR = 1.58, *p* = 0.007) and SII (OR = 1.57, *p* = 0.032) continued to be independent predictors. The TSAT–cholestatic association was preserved in the same direction but did not reach significance in this smaller subgroup. When ferritin was removed from the multivariable model, the principal cholestatic associations (HbA1c and SII) were preserved, and TSAT emerged as an independent correlate of the hepatocellular pattern (Supplementary Table S2). When the internal validity of the cholestatic full model was assessed by non-parametric bootstrap with 1,000 iterations, the empirical 95% confidence intervals for HbA1c, SII, and TSAT excluded 1.00, and the bootstrap median ORs remained consistent with the original point estimates (Supplementary Table S3). Pattern-specific post-hoc statistical power, estimated under the Hsieh approximation, was approximately 73% for HbA1c, 99% for SII, and 80% for TSAT in the cholestatic full model (*n* = 625, events = 77), and approximately 92% for ferritin in the hepatocellular full model (*n* = 653, events = 105).

In an additional pre-specified sensitivity analysis, HbA1c was residualised against age using linear regression (*β*₁ = 0.028% per year; *R*^2^ = 0.083; *p* < 0.001), and the age-residualised HbA1c was entered in place of raw HbA1c into the cholestatic multivariable model. The association with the cholestatic pattern was preserved with virtually unchanged magnitude (OR = 1.551; 95% CI: 1.157–2.079; *p* = 0.003 with age retained as a separate covariate; OR = 1.462; 95% CI: 1.098–1.946; *p* = 0.009 with age omitted). Coefficients for SII (OR = 2.177; 95% CI: 1.221–3.881), TSAT (OR = 0.665; 95% CI: 0.470–0.940), and ferritin (OR = 1.391; 95% CI: 0.994–1.946) were unchanged in the model with age retained, confirming that the HbA1c–cholestatic association is not attributable to age confounding.

As a further pre-specified sensitivity analysis, the multivariable models were repeated stratified by MASLD status. In the MASLD-positive cholestatic subset (*n* = 351, events = 45), the cholestatic pattern remained independently associated with HbA1c (OR = 1.40; 95% CI: 1.06–1.86; *p* = 0.020), TSAT (OR = 0.60; 95% CI: 0.39–0.94; *p* = 0.024), and SII (OR = 1.59; 95% CI: 1.03–2.47; *p* = 0.039). In the MASLD-negative cholestatic subset (*n* = 274, events = 32), HbA1c (OR = 1.59; *p* = 0.006) and SII (OR = 1.58; *p* = 0.029) remained independent, while the TSAT association attenuated (OR = 0.76; *p* = 0.281) in this smaller subset. For the hepatocellular pattern, the ferritin association was preserved in the MASLD-negative subset (*n* = 295, events = 53; OR = 2.14; 95% CI: 1.45–3.16; *p* < 0.001) and attenuated in the MASLD-positive subset (OR = 1.32; *p* = 0.085).

### 3.4 Multivariable direct comparison

In the direct comparison between the two borderline patterns (*n* = 182), the full multivariable model identified TSAT (OR = 0.532; 95% CI: 0.323–0.877; *p* = 0.013), SII (OR = 7.082; 95% CI: 1.336–37.527; *p* = 0.021), and HbA1c (OR = 2.049; 95% CI: 1.100–3.817; *p* = 0.024) as independent variables distinguishing the cholestatic pattern from the hepatocellular pattern (EPV = 4.5). In the reduced model (EPV = 9.6), TSAT (OR = 0.565, *p* = 0.020), SII (OR = 4.445, *p* = 0.002), and HbA1c (OR = 1.484, *p* = 0.040) remained consistent with the findings of the full model; in addition, age (OR = 1.585, *p* = 0.029) emerged as a further independent discriminator in this model. All VIF values were <3.1 (Table 4).

**Table 4:** Multivariable direct comparison: cholestatic versus hepatocellular pattern (*n* = 182).

Variable	Full model OR (95% CI)	*p*	Reduced model OR (95% CI)	*p*	VIF (full)
HbA1c	2.049 (1.100–3.817)	0.024*	1.484 (1.018–2.164)	0.040*	2.5
TSAT	0.532 (0.323–0.877)	0.013*	0.565 (0.349–0.915)	0.020*	1.6
SII	7.082 (1.336–37.527)	0.021*	4.445 (1.759–11.236)	0.002**	3.0
Age	1.571 (0.850–2.902)	0.149	1.585 (1.049–2.393)	0.029*	2.8
Female sex	0.799 (0.286–2.230)	0.668	0.827 (0.320–2.134)	0.694	2.0
Ferritin	0.959 (0.584–1.575)	0.870	0.889 (0.565–1.398)	0.610	1.8
BMI	1.382 (0.926–2.062)	0.114	1.377 (0.930–2.040)	0.108	1.4
CRP	1.410 (0.793–2.507)	0.242	1.350 (0.777–2.346)	0.287	2.0
TG/HDL ratio	0.880 (0.574–1.349)	0.557	–	–	1.1
NLR	0.793 (0.322–1.956)	0.615	–	–	2.7
NMR	0.990 (0.694–1.412)	0.956	–	–	1.1
eGFR	1.015 (0.552–1.866)	0.962	–	–	2.7
Albumin	0.855 (0.560–1.304)	0.467	–	–	1.3

Reference category: hepatocellular pattern. Primary parsimonious model (reduced): *n* = 182, EPV = 9.6; 8 pre-specified variables (HbA1c, TSAT, SII, sex, age, BMI, ferritin, CRP). Sensitivity model (full): *n* = 182, EPV = 4.5 (17 variables); findings hypothesis-generating given EPV<5, particularly the SII estimate whose wide 95% CI (1.336–37.527) precludes precise effect-size inference. Both models additionally adjusted for alcohol use, steatosis grade, DM diagnosis, and hepatotoxic drug use. Continuous variables standardised; ORs expressed per 1 SD increment. HbA1c, Glycated haemoglobin; TSAT, Transferrin saturation; SII, Systemic immune-inflammation index; BMI, Body mass index; CRP, C-reactive protein; TG/HDL, Triglyceride-to-HDL cholesterol ratio; NLR, Neutrophil-to-lymphocyte ratio; NMR, Neutrophil-to-monocyte ratio; eGFR, Estimated glomerular filtration rate; DM, Diabetes mellitus; OR, Odds ratio; CI, Confidence interval; VIF, Variance inflation factor. *p < 0.05; *p < 0.01.

### 3.5 Fibrosis assessment

The median FIB-4 value was <1.0 across all groups (normal, hepatocellular, cholestatic, overlap), and ≥82% of participants in each group fell within the low-risk category (age-adjusted FIB-4 < 1.3). No significant between-group difference was observed (Kruskal–Wallis *p* = 0.72). APRI values also remained below the 0.5 threshold recommended for advanced fibrosis across all groups (Supplementary Table S4).

### 3.6 Overlap pattern

The overlap group (*n* = 29; 69.0% female) exhibited features of both pure patterns: ferritin was elevated (median 52.0 μg/L, *p* = 0.036 vs. normal) and HbA1c was higher (6.60% vs. 5.70%, *p* < 0.001), whereas CRP did not differ significantly from the normal group (*p* = 0.428).

### 3.7 Steatosis and metabolic parameters

Hepatic steatosis was present in 57.8% of the borderline elevation cohort. Across the entire cohort, HbA1c, BMI, triglycerides, and fasting glucose increased progressively with steatosis grade (all Kruskal–Wallis *p* < 0.05), whereas NLR and CRP showed no significant gradient (Table 5). The proportion of patients with borderline enzyme elevation was broadly similar across steatosis grades 0–2 (25.2–26.7%); a numerically higher rate (42.9%) was observed in Grade 3, but this did not reach statistical significance (*p* = 0.360; *n* = 21).

**Table 5:** Steatosis grade and key metabolic/inflammatory parameters (*n* = 800).

Variable	Absent (*n* = 333)	Grade 1 (*n* = 290)	Grade 2 (*n* = 156)	Grade 3 (*n* = 21)	*p* (KW)
HbA1c (%)	5.6 (5.3–6.1)	5.8 (5.4–6.6)	5.9 (5.4–6.7)	6.2 (5.6–6.7)	0.001
BMI (kg/m^2^)	27.4 (25.0–30.2)	28.5 (25.7–31.6)	29.3 (27.1–32.3)	30.2 (28.5–31.1)	<0.001
Triglycerides (mg/dL)	128 (89–206)	142 (99–217)	146 (96–203)	204 (164–268)	0.013
Fasting glucose (mg/dL)	95 (87–113)	98 (87–125)	98 (86–123)	112 (97–143)	0.011
TG/HDL ratio	2.6 (1.6–4.3)	2.9 (1.9–4.9)	2.9 (1.8–4.4)	4.1 (2.4–5.9)	0.026
NLR	1.8 (1.4–2.3)	1.9 (1.5–2.5)	1.8 (1.5–2.4)	1.8 (1.4–3.0)	0.409
CRP (mg/L)	3.0 (1.8–5.8)	3.0 (1.7–5.3)	3.3 (1.7–5.6)	2.7 (2.0–6.7)	0.849
Borderline elevation	89/333 (26.7%)	73/290 (25.2%)	40/156 (25.6%)	9/21 (42.9%)	0.360

Values: median (IQR) or *n* (%). KW, Kruskal–Wallis test; HbA1c, Glycated haemoglobin; BMI, Body mass index; TG/HDL, Triglyceride-to-HDL cholesterol ratio; NLR, Neutrophil-to-lymphocyte ratio; CRP, C-reactive protein.

## 4 Discussion

This observational outpatient study, based on standardised repeat measurements, demonstrates that persistent borderline liver enzyme elevation — falling between one and two times the sex-specific ULN — carries biochemically distinguishable signatures according to pattern. The hepatocellular and cholestatic patterns emerge with different biochemical profiles, revealing a biological divergence that may go unnoticed when the borderline range is treated as a single category.

One of the clinically most notable findings of our study is that the cholestatic pattern is characterised not by a single marker but by a co-clustered metabolic–inflammatory profile. The three variables independently associated with this pattern — elevated HbA1c, elevated SII, and reduced TSAT — reflect different facets of the same pathophysiological picture: glycaemic burden, systemic inflammation, and inflammation-mediated iron metabolism disturbance. The most striking aspect of these associations is that HbA1c is linked to the cholestatic pattern, whereas no such association is observed with the hepatocellular pattern. Moreover, this link was preserved in non-diabetic patients, and age did not emerge as an independent determinant in the same model. This suggests that the association relates more to glycaemic burden itself than to an established diagnosis of diabetes.

The relationship between glycaemic dysregulation and liver abnormality has been examined predominantly through the lens of steatotic liver disease: NAFLD/MASLD prevalence is high in patients with T2D (14, 15), histological severity increases progressively with HbA1c (16), and this relationship has also been demonstrated in non-diabetic adults through HbA1c-based composite markers (17, 18). However, most of the existing literature characterises liver involvement on the basis of imaging- or biopsy-defined steatosis, without differentiating by enzyme pattern. By contrast, GGT, the principal marker of the cholestatic range, has been identified in large-scale cohorts as an independent predictor of type 2 diabetes, hypertension, and metabolic syndrome even before the development of established diabetes; in particular, cumulative high GGT exposure increases diabetes risk in a dose–response manner (19, 20). Within this framework, our finding — that the borderline cholestatic pattern remains independently associated with HbA1c even in the absence of overt disease and outside established diabetes — indicates that the imprint of glycaemic burden on the liver is clearly traceable not only along the steatotic axis but also along the cholestatic axis at subthreshold levels. This inference is further strengthened by the absence of a significant HbA1c association for the hepatocellular pattern in the same cohort; the glycaemic signal is therefore not a phenomenon independent of enzyme pattern but one that accumulates predominantly along the cholestatic axis.

The second dimension is the low-grade systemic inflammation accompanying metabolic burden and its reflection on iron metabolism. The association between composite inflammatory indices and NAFLD/MAFLD has been consistently demonstrated both in large prospective cohorts (UK Biobank, *n* = 378,139) and in multicentre Chinese cohorts (21, 22); NLR has been linked to all-cause and cardiovascular mortality in the setting of MASLD (23), and has been identified as an independent determinant of MASLD in the type 2 diabetes subpopulation (24). However, this literature has not disentangled which enzyme pattern inflammatory indices specifically cluster with, and the findings have mostly been reported in the context of overt disease or a diagnosis of steatosis. In our cohort, the fact that SII was independently associated only with the cholestatic pattern, while inflammatory indices in the hepatocellular group were even lower than in the normal group, suggests that the inflammatory signal within the borderline range is not homogeneous and may be specific to one subphenotype. A finding that completes this picture is the concomitantly reduced TSAT in the cholestatic group: in the setting of inflammation, interleukin-6-mediated upregulation of hepcidin suppresses iron release via ferroportin, thereby lowering serum iron and TSAT — a well-defined mechanism now regarded as a key regulator of iron homeostasis in obesity-related metabolic disorders (25). Supporting this interpretation at the analytical level, a significant negative correlation between TSAT and SII was observed within the cholestatic group (Spearman rho = −0.299, *p* = 0.008); this indicates that iron transport saturation decreases as inflammatory burden increases, quantitatively strengthening the hypothesis that the reduced TSAT reflects functional iron restriction on a background of chronic inflammation. As a symmetric within-group test in the hepatocellular pattern, ferritin showed no correlation with CRP (Spearman rho = −0.010, *p* = 0.921) and a weak inverse correlation with SII (rho = −0.201, *p* = 0.040); neither direction is compatible with an acute-phase-driven ferritin elevation. Together with the group-level inflammatory indices being lower than in the normal group, this provides a formal counterpart to the TSAT–SII correlation reported above for the cholestatic group and reinforces the interpretation of the hepatocellular iron signature as consistent with the metabolic hyperferritinaemia spectrum rather than a reflection of systemic inflammation. To formally examine whether this reduced TSAT reflects true iron deficiency, functional iron restriction, or anaemia of chronic disease, haematological and iron-handling parameters were compared. Haemoglobin, haematocrit, and total iron-binding capacity did not differ from the normal group (Hb 14.3 vs. 14.4 g/dL, *p* = 0.400; TIBC 356 vs. 356 μg/dL, *p* = 0.405), and the prevalence of WHO-defined anaemia was comparable (9.1% vs. 7.1%, *p* = 0.698), rendering classical anaemia of chronic disease an unlikely unifying explanation. MCV was modestly lower (84.7 vs. 86.4 fL, *p* = 0.031) while remaining within the normocytic range. Within the cholestatic group, 32.5% met criteria for absolute iron deficiency (ferritin < 30 μg/L and TSAT < 20%) and 23.4% exhibited functional iron restriction without anaemia (low TSAT with preserved ferritin and haemoglobin), whereas only one patient (1.3%) met the classical ACD pattern. The cholestatic reduced-TSAT signal therefore reflects a composite of absolute iron depletion and inflammation-associated functional restriction rather than ACD; the hepcidin-mediated interpretation applies to the latter subset, while the former would warrant separate clinical evaluation for absolute iron depletion. The co-clustering of HbA1c, SII, and reduced TSAT suggests that the cholestatic phenotype is aligned with a shared pathophysiological axis defined by the triad of glycaemic burden, low-grade inflammation, and inflammation-mediated iron sequestration; in contrast, the oppositely directed iron markers of the hepatocellular pattern (elevated ferritin, serum iron, and TSAT) indicate that these two phenotypes diverge not only in their enzyme profile but also in their iron metabolism. The wide confidence interval of the SII estimate in the full direct-comparison model (EPV = 4.5) indicates that this effect-size estimate should be viewed as hypothesis-generating, although the directional association was preserved in the reduced model and in bootstrap validation.

An important clinical signature of the cholestatic pattern is its marked female predominance (70% vs. 57% in the normal group). This finding is consistent with the general pattern observed in the MASLD literature: during the premenopausal period, oestrogen-mediated protection relatively slows the development of steatotic and inflammatory liver disease in women, whereas after menopause, with the withdrawal of this protection, the incidence of the disease equalises with that in men, and the risk of advanced fibrosis and steatohepatitis has even been reported to become more pronounced in women (10). This clinical observation is also mechanistically supported by the fact that the liver is one of the organs exhibiting sexual dimorphism and that sex hormones drive liver-specific gene expression and metabolic activity (9). In our cohort, the fact that the cholestatic group was significantly older than the hepatocellular group (median 56 vs. 46 years) complements this picture, conveying an impression compatible with a metabolic–inflammatory transition pattern corresponding to the postmenopausal age range. Within the cholestatic group, 72% (39/54) of the women were aged ≥ 50 years and 52% (28/54) were ≥ 55 years, compared with 45% aged ≥ 50 years in the normal female subgroup and 50% in the hepatocellular female subgroup. Although menopausal status was not directly ascertained, this age distribution places the majority of cholestatic women within the age range in which postmenopausal transition is typical.

This biological background is also important for the interpretation of the observed HbA1c and TSAT findings. The post-menopausal decline in oestrogen may contribute to the higher HbA1c levels observed in the cholestatic group through a reduction in insulin sensitivity. Conversely, while the cessation of menstruation and the subsequent replenishment of iron stores would be expected to raise TSAT, the lower TSAT observed in the cholestatic group in our cohort suggests that this finding cannot be explained by menopausal physiology alone; rather, it makes hepcidin-mediated functional iron restriction, on a background of the systemic inflammation reflected by the concomitantly elevated SII, the more likely explanation. A retrospective cross-check of the e-Prescription and Central Physician Information System records identified no active hormone replacement therapy prescription among the female participants; HRT-mediated modification of the glycaemic and iron signals is therefore unlikely to account for the observed associations.

An important distinction should be made at this point: the pre-specified sex × covariate interaction tests were non-significant in both the cholestatic–normal and the direct-comparison models. That is, female predominance and older age are compositional features defining the cholestatic phenotype; there is no evidence that the association between HbA1c, SII, and TSAT with the pattern is modified by sex. This suggests that our observation is not an artefact arising solely from sex composition; rather, the borderline cholestatic pattern represents a genuine phenotype, grounded in a shared cardiometabolic–inflammatory background, that emerges prominently in postmenopausal women. The hepatocellular pattern, by contrast, diverges markedly from this picture and is situated on a different pathophysiological axis.

The second prominent finding of our study is that the hepatocellular pattern diverges markedly from the cholestatic pattern not only in its enzyme profile but also in its underlying biological signature. The only variable independently associated with this pattern is ferritin, and the concomitant elevation of serum iron and TSAT points to an iron-related biochemical signature rather than a picture secondary to inflammation. The most striking aspect of these relationships is the absence of the glycaemic and inflammatory signals seen in the cholestatic pattern; inflammatory indices (SII, NLR, NMR) in the hepatocellular group were even lower than in normal controls. Moreover, this pattern shows no sex predominance, and the group has a younger age profile compared with the cholestatic group. This suggests that the hepatocellular pattern represents a phenotype that is independent of the metabolic–inflammatory axis, aligned more closely with the iron metabolism axis, and represents a patient profile distinct from the cholestatic pattern.

The emergence of ferritin as the only independent determinant of the hepatocellular pattern closely aligns with the concept of metabolic hyperferritinaemia in the literature. In the current consensus statement, this entity is defined as elevated serum ferritin levels on a background of metabolic dysfunction, accompanied by mild-to-moderate iron deposition in the reticuloendothelial system, and is associated with increased cardiometabolic and liver-related risk (26). In large MASLD cohorts, metabolic hyperferritinaemia has been shown to be independently associated with both all-cause mortality and liver-related event risk (27); pathophysiologically, this entity is explained by an inadequate hepcidin response in the setting of insulin resistance, resulting in increased iron accumulation (25, 26). A low AST/ALT ratio has also been defined as a typical biochemical indicator of ALT predominance in the early stage of MASLD (28), and recent findings that biochemical patterns can differentiate MASLD subphenotypes and prognosis further reinforce the clinical value of this approach (29).

In our cohort, the concomitant elevation of ferritin, serum iron, and TSAT against a background in which composite inflammatory indices (SII, NLR, NMR) were even lower than in the normal group and CRP did not differ from normal (*p* = 0.133) indicates that the signal is more consistent with an iron-related biochemical signature than with an acute-phase response, and that it fits conceptually within the metabolic hyperferritinaemia spectrum. Supporting this interpretation at the analytical level, within the hepatocellular group ferritin was not significantly correlated with CRP (Spearman rho = −0.010, *p* = 0.921) and showed only a weak inverse correlation with SII (rho = −0.201, *p* = 0.040), whereas it correlated strongly with other iron-metabolism markers (TSAT rho = +0.546, *p* < 0.001; serum iron rho = +0.466, *p* < 0.001). This pattern — ferritin dissociated from inflammatory indices and tightly co-clustered with iron-transport parameters — parallels, in the opposite direction, the TSAT–SII coupling observed in the cholestatic group and reinforces the interpretation that the hepatocellular ferritin elevation reflects an iron-loading signature rather than an acute-phase response. Within this framework, the original contribution of our study lies in demonstrating that this iron-predominant phenotype can be identified as a specific biochemical signature for the hepatocellular pattern beneath the threshold of overt disease — within the borderline range — when delineated using sex-specific ULN.

Although the concomitant elevation of creatine kinase and LDH observed in this group raises the possibility of an extrahepatic muscle contribution, this contribution cannot be entirely excluded, as recent exercise, history of muscle injury, baseline physical activity level, and long-term stable statin use were not systematically recorded. However, whereas borderline myopathy typically produces an AST-predominant enzyme pattern (7), the picture observed in this study is ALT-predominant with a low AST/ALT ratio; furthermore, muscle-derived enzyme release does not carry an iron signal such as elevated ferritin and TSAT (26). It is therefore unlikely that the observed biochemical signature could be entirely accounted for by an extrahepatic source; nevertheless, the possibility that an unrecognised subgroup with asymptomatic myopathy may have been present within the group and may have contributed to heterogeneity in its biochemical profile should not be dismissed. The absence of more tissue-specific markers such as aldolase or 5′-nucleotidase from the routine panel limits this distinction; similarly, in the cholestatic pattern, the inability to differentiate non-hepatic ALP isoforms is a factor that constrains specificity (8). For this reason, the “hepatocellular” and “cholestatic” designations in our findings should be regarded as operational distinctions achievable at the level of the standard biochemistry panel, and do not carry a claim of definitive tissue-level liver specificity.

Another factor that may influence the observed pattern distribution is the long-term stable medications in common use that do not cause overt hepatotoxicity but can nonetheless produce borderline enzyme changes. Statins are known to exert effects along the hepatocellular axis through mild ALT elevation (30), whereas oral hormone replacement therapy and certain preparations containing anabolic/androgenic agents have established effects along the cholestatic axis (31); long-term NSAIDs and certain antihypertensives may also rarely affect enzyme levels. In our study, hepatotoxic medications initiated or modified within the preceding 4 weeks were applied as an exclusion criterion, hepatotoxic drug use was included as a covariate in the multivariable models, and oral contraceptive use was retained as a separate exclusion criterion (Methods 2.1 and 2.3). Nevertheless, because the mild enzyme effects of long-term stable therapies were not systematically recorded, the possibility that these medications may have contributed to pattern assignment in a subgroup lying at the borders of either the hepatocellular or the cholestatic pattern cannot be excluded. Particularly when considered together with the older age profile of the cholestatic group, in which polypharmacy increases with age, this should be regarded as a residual confounder to be taken into account when interpreting the findings.

In the hepatocellular group, in addition to iron markers, albumin and haematocrit were also mildly elevated (44 vs. 43 g/L, *p* = 0.006; and 43.1% vs. 42.1%, *p* = 0.035, respectively); BUN, however, did not differ (*p* = 0.702). This picture weakens the likelihood of systemic haemoconcentration or dehydration, and the failure of these differences to reach independent significance in the multivariable model suggests that the finding reflects compositional features of the group rather than a pathological process. The clinical value of biochemical pattern-based phenotyping has recently been demonstrated more explicitly: in the HEPAmet registry study of 2,156 biopsy-confirmed MASLD patients, the cholestatic pattern was associated with more cirrhosis and an increased all-cause mortality (HR 2.37) (29). While some alignment with that study is observed — the association of the cholestatic pattern with older age conveys a similar impression in both cohorts — the direction of the metabolic accompaniments differs: whereas that study linked the hepatocellular pattern with higher glucose and lipid levels, in our cohort the glycaemic and metabolic–inflammatory burden accumulates predominantly along the cholestatic axis. Two potential sources account for this difference: (i) population selection — a cohort of outpatients with borderline enzyme elevation, most of whom remain below the threshold of overt disease, contrasted with biopsy-confirmed patients largely at the stage of overt MASLD; and (ii) pattern definition — a classification based on whether each individual enzyme exceeds its sex-specific ULN, as opposed to an enzyme-ratio-based classification (ALT/ULN to ALP/ULN). Taken together with these methodological differences, the two approaches can be regarded as complementary rather than conflicting: biochemical pattern carries value as a prognostic discriminator in the setting of overt disease, while in the borderline range it can define clinically meaningful subphenotypes.

The overlap group (*n* = 29) exhibited the highest HbA1c among all borderline subgroups (6.60%, *p* < 0.001 vs. normal) and elevated ferritin levels (52.0 μg/L, *p* = 0.036), thereby combining the glycaemic–inflammatory signal of the cholestatic pattern with the iron-loading feature of the hepatocellular pattern. This combined signature was accompanied by the highest proportion of participants meeting MASLD criteria across all groups (72.4%) and a marked female predominance (69%); the fact that CRP did not differ from the normal group (*p* = 0.428) suggests that this picture is not driven by an acute-phase response. Taken together, these findings give the impression that the overlap group may represent a metabolically more burdened phenotype than either pure pattern alone. Nevertheless, owing to the limited sample size (*n* = 29), this subgroup was not included in the multivariable regression analyses, and the univariable comparisons involving this group carry limited precision with correspondingly wide confidence intervals. For this reason, the findings pertaining to the overlap group should be regarded as hypothesis-generating rather than confirmatory, and warrant testing in larger cohorts (3).

Expected metabolic gradients were observed across steatosis grades (progressive increases in HbA1c, BMI, triglycerides, and fasting glucose; *p* < 0.05); however, steatosis grade did not independently account for pattern assignment in any of the multivariable models. Similarly, FIB-4 and APRI values did not differ significantly between groups, suggesting that there is no notable fibrosis signal in individuals with borderline enzyme elevation irrespective of pattern. Taken together, these two findings indicate that the biochemical signatures of the hepatocellular and cholestatic patterns cannot be directly accounted for by either advanced steatosis or advanced fibrosis. This interpretation is further supported by the stable preservation of the principal findings (the HbA1c–cholestatic and ferritin–hepatocellular associations) in the subcohort without steatosis; accordingly, the divergence between patterns can be said to emerge on a systemic metabolic–inflammatory background independent of hepatic fat burden and fibrosis. The numerically higher prevalence of borderline elevation observed in Grade 3 steatosis (42.9% vs. approximately 26% in grades 0–2) did not reach statistical significance owing to the small group size (*n* = 21).

When the multivariable models were stratified by MASLD status, the core cholestatic signature — independent associations of HbA1c and SII — was preserved in both MASLD-positive and MASLD-negative subsets, indicating that the glycaemic–inflammatory axis co-clusters with the cholestatic pattern irrespective of MASLD status; the TSAT association attenuated in the smaller MASLD-negative subset. Notably, the hepatocellular–ferritin association was substantially stronger in the MASLD-negative subset than in the MASLD-positive subset, supporting the interpretation that the iron-predominant signature of the borderline hepatocellular pattern aligns with the metabolic hyperferritinaemia spectrum and operates in part independently of steatosis. These findings indicate that the two pattern-specific signatures are not confined to the MASLD subpopulation.

**Figure 2:**
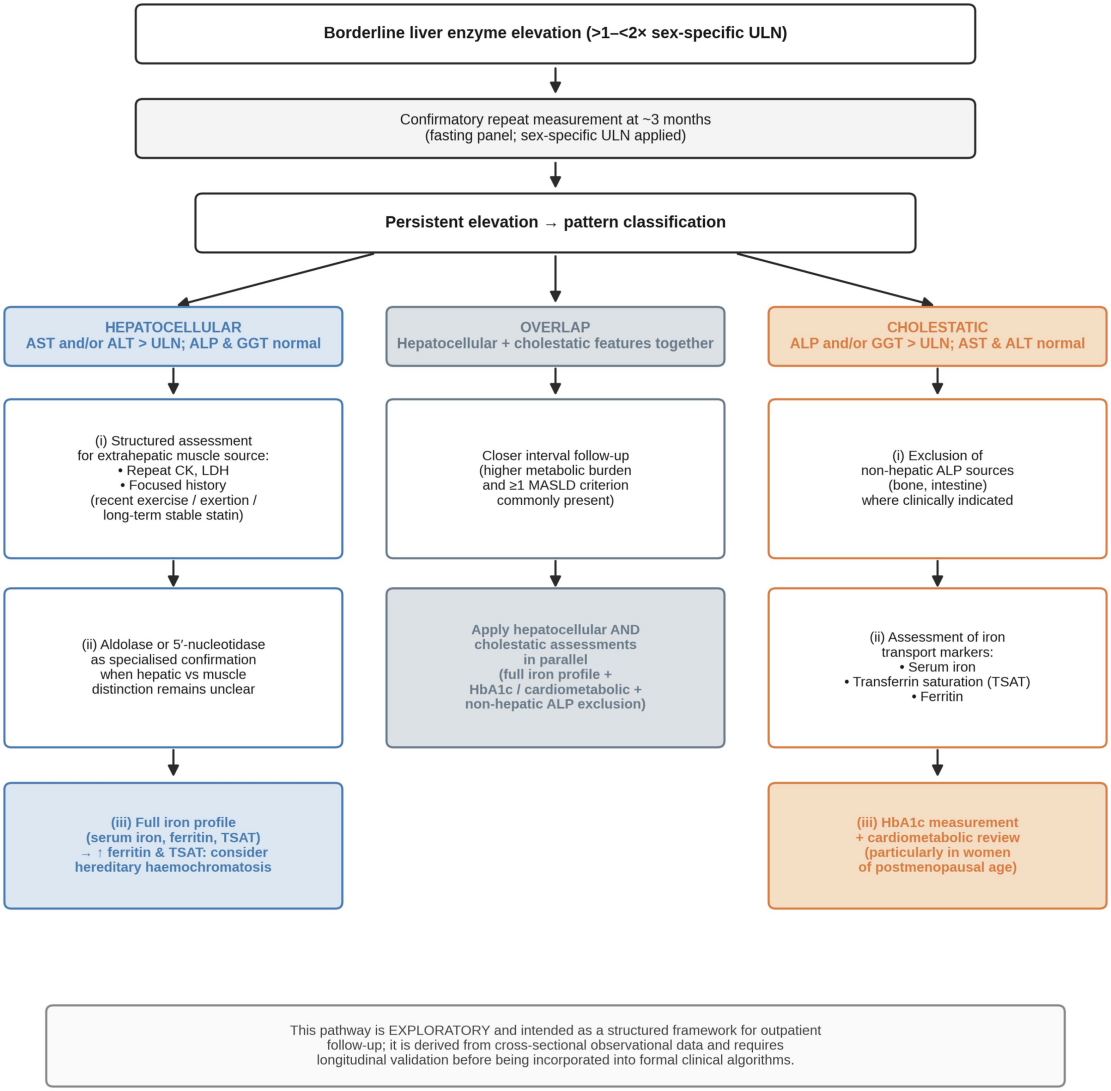
Proposed pattern-specific evaluation pathway for persistent borderline liver enzyme elevation. Once borderline elevation (>1–<2 × sex-specific ULN) is confirmed on repeat measurement, cases are stratified into hepatocellular, cholestatic, or overlap patterns, each mapped to a stepwise workup anchored on the biochemical signatures identified in this study: an iron-predominant signature in the hepatocellular pattern (with ferritin as the only independent correlate) and a glycaemic–inflammatory–reduced-iron-transport signature in the cholestatic pattern (HbA1c, SII, and TSAT independently associated). The pathway is exploratory and derived from cross-sectional observational data; longitudinal validation is required before incorporation into formal clinical algorithms.

In conclusion, borderline liver enzyme elevation persisting over 3 months represents two biochemically distinct phenotypes when sex-specific reference ranges are applied. The cholestatic pattern is female-predominant and shows independent associations with glycaemic burden (HbA1c), reduced iron transport (TSAT), and systemic inflammatory activation (SII); this pattern is preserved even in non-diabetic patients. The hepatocellular pattern, in contrast, is characterised by elevated iron markers (ferritin, serum iron, and TSAT), without sex predominance or inflammatory activation, and exhibits a biochemical signature consistent with the metabolic hyperferritinaemia spectrum. These findings suggest that pattern recognition using sex-appropriate thresholds may carry practical clinical value in the outpatient follow-up setting. Pattern-specific suggestions derived from these findings are summarised in Figure 2. These suggestions are exploratory in nature and require longitudinal validation before being incorporated into formal algorithms.

## 5 Limitations

The observational design limits causal inference, and as no structured diagnostic work-up was performed, residual confounding from unidentified aetiologies cannot be excluded. Since the baseline characteristics of the 151 patients lost to follow-up (13.4%) could not be compared with those of the analysed cohort, attrition bias cannot be fully excluded. The cohort was recruited at a single tertiary internal medicine outpatient clinic in eastern Türkiye, and the findings therefore reflect the referral patterns and metabolic profile of this region, with referral bias remaining a structural limitation. As genetic variants with well-established effects on liver enzymes and susceptibility to steatosis (particularly PNPLA3 rs738409) were not assessed, the generalisability of the findings to populations with different genetic backgrounds is limited. Hepatic steatosis was graded by abdominal ultrasonography, which has known limitations including low sensitivity for mild steatosis, operator dependency, and the inability to assess fibrosis; CAP or MRI-PDFF could have provided higher accuracy. The absence of histological confirmation and genetic testing for hereditary iron disorders constrains the aetiological interpretation of the iron-related findings. Menopausal status, hormone replacement therapy, insulin resistance markers, waist circumference, dietary and physical activity data, and mechanistic iron-regulatory markers (hepcidin, inflammatory cytokines) were not assessed; nor were the mild enzyme effects of long-term stable therapies (statins, NSAIDs, hormone replacement) systematically recorded, and residual confounding attributable to these variables cannot be excluded. Finally, the study does not include clinical outcome data such as progression to overt liver disease, fibrosis development, incident diabetes, cardiovascular events, and mortality. Although the three-month follow-up confirms persistent enzyme elevation, long-term longitudinal studies are required to establish the stability of the patterns over time and their prognostic value. The three-month interval applied here was designed to confirm persistent enzyme elevation rather than to characterise long-term pattern stability. Ampuero et al. reported that most patients maintained their initial biochemical pattern at a second evaluation in the HEPAmet Registry (29), implying that a subset does shift pattern over time; whether borderline-range patients exhibit similar longitudinal stability, or are more prone to pattern transition, cannot be addressed by our design and requires future longitudinal cohorts with serial biochemical classification.

## Data Availability

The raw data supporting the conclusions of this article will be made available by the authors, without undue reservation.
