# Supplementary material for: Borderline liver enzyme patterns and their metabolic–inflammatory signatures: an observational outpatient study
Source: Front Med (Lausanne). 2026 May 15;13:1838167. doi: 10.3389/fmed.2026.1838167 (PMC13218902; doi:10.3389/fmed.2026.1838167)
Supplement: Supplementary file 1 [file Data_Sheet_1.DOCX]

**SUPPLEMENTARY MATERIALS**

**Supplementary Table S1.** Baseline characteristics of the overt elevation group (n = 41, ≥ 2 × ULN), with comparisons to the normal and borderline groups

| **Variable** | **Overt (n=41)** | **Normal (n=548)** | **Borderline (n=211)** | **p†** | **p‡** |
| --- | --- | --- | --- | --- | --- |
| Age (years) | 48.0 (39–54) | 50.0 (38–63) | 52.0 (41–62) | 0.541 | 0.247 |
| Female sex, n (%) | 19 (46.3%) | 310 (56.6%) | 130 (61.6%) | 0.267 | 0.100 |
| BMI (kg/m²) | 28.3 (26–31) | 28.4 (26–31) | 29.0 (25–32) | 0.962 | 0.498 |
| HbA1c (%) | 5.60 (5.30–6.10) | 5.70 (5.30–6.30) | 5.90 (5.50–7.20) | 0.749 | 0.026* |
| TG/HDL ratio | 3.10 (2.38–4.80) | 2.60 (1.70–4.50) | 3.10 (1.80–4.70) | 0.071 | 0.412 |
| Triglycerides (mg/dL) | 158 (123–267) | 131 (91–204) | 163 (99–227) | 0.017* | 0.370 |
| Total cholesterol (mg/dL) | 193 (165–226) | 187 (159–214) | 202 (170–230) | 0.240 | 0.508 |
| Fasting glucose (mg/dL) | 97 (89–118) | 96 (86–115) | 101 (89–148) | 0.956 | 0.123 |
| Ferritin (μg/L) | 66.0 (29–116) | 34.0 (15–64) | 45.0 (20–90) | <0.001** | 0.071 |
| TSAT (%) | 24.9 (18.6–32.5) | 21.8 (15.3–29.8) | 20.7 (13.5–29.1) | 0.095 | 0.045* |
| AISI | 0.36 (0.22–0.59) | 0.13 (0.07–0.26) | 0.13 (0.07–0.26) | 0.532 | 0.512 |
| NLR | 1.72 (1.40–2.73) | 1.85 (1.48–2.38) | 1.82 (1.48–2.42) | 0.655 | 0.559 |
| NMR | 7.97 (5.96–8.99) | 7.70 (6.24–9.28) | 7.83 (6.31–9.51) | 0.745 | 0.924 |
| CRP (mg/L) | 3.80 (2.00–6.60) | 3.20 (1.80–5.40) | 2.60 (1.60–5.80) | 0.136 | 0.093 |
| Albumin (g/L) | 43.0 (41–45) | 43.0 (41–45) | 43.0 (41–46) | 0.896 | 0.375 |
| Haemoglobin (g/dL) | 14.7 (14–16) | 14.4 (13–16) | 14.6 (14–16) | 0.062 | 0.269 |
| HCT (%) | 43.0 (41–47) | 42.1 (40–45) | 42.8 (40–46) | 0.109 | 0.493 |
| eGFR (mL/min/1.73m²) | 107 (99–118) | 100 (89–113) | 102 (90–115) | 0.190 | 0.328 |
| Hepatic steatosis, n (%) | 24 (58.5%) | 321 (58.6%) | 122 (57.8%) | 1.000 | 1.000 |
| Smoking, n (%) | 8 (19.5%) | 188 (34.3%) | 70 (33.2%) | 0.077 | 0.122 |
| Alcohol use, n (%) | 7 (17.1%) | 63 (11.5%) | 21 (10.0%) | 0.416 | 0.291 |
| Diabetes mellitus, n (%) | 6 (14.6%) | 132 (24.1%) | 72 (34.1%) | 0.235 | 0.022* |

*†Overt vs Normal; ‡Overt vs Borderline. Continuous variables: median (IQR), Mann–Whitney U test. Categorical variables: n (%), Pearson chi-square or Fisher exact test as appropriate. *p < 0.05; **p < 0.01. ≥ 2×ULN is defined as any of AST, ALT, GGT, or ALP exceeding twice the sex-specific upper limit of normal. BMI: body mass index; HbA1c: glycated haemoglobin; TG/HDL: triglyceride-to-HDL cholesterol ratio; TSAT: transferrin saturation; AISI: aggregate index of systemic inflammation; NLR: neutrophil-to-lymphocyte ratio; NMR: neutrophil-to-monocyte ratio; CRP: C-reactive protein; HCT: haematocrit; eGFR: estimated glomerular filtration rate.*

**Supplementary Table S2.** Multivariable logistic regression in the steatosis-free subcohort (pre-specified reduced model).

| Model (n / events / EPV) | Variable | OR (95% CI) | p |
| --- | --- | --- | --- |
| Hepatocellular vs Normal (277 / 50 / 6.2) | Ferritin | 2.57 (1.61–4.13) | <0.001 |
| Cholestatic vs Normal (258 / 31 / 3.9) | HbA1c | 1.58 (1.13–2.20) | 0.007 |
|  | SII | 1.57 (1.04–2.38) | 0.032 |
|  | TSAT | 0.83 (0.48–1.43) | 0.493 |
|  | Ferritin | 1.59 (0.95–2.67) | 0.079 |

*Reduced multivariable model (HbA1c, TSAT, ferritin, SII, sex, age, BMI, CRP) in participants without ultrasound-detected hepatic steatosis. Continuous variables are standardised; ORs expressed per 1 SD increment. OR: odds ratio; CI: confidence interval; EPV: events per variable; HbA1c: glycated haemoglobin; TSAT: transferrin saturation; SII: systemic immune-inflammation index; BMI: body mass index; CRP: C-reactive protein.*

**Supplementary Table S3.** Bootstrap internal validation of the cholestatic full model (1000 iterations).

| Variable | Original OR | Bootstrap median OR | Bootstrap 95% CI |
| --- | --- | --- | --- |
| HbA1c | 1.64 | 1.66 | 1.26–2.31 |
| SII | 1.77 | 1.80 | 1.31–2.68 |
| TSAT | 0.67 | 0.66 | 0.45–0.92 |
| Ferritin | 1.36 | 1.38 | 0.98–2.04 |

*Non-parametric bootstrap with 1000 resamples. Continuous variables are standardised; ORs expressed per 1 SD increment. Bootstrap median ORs were within 2% of original point estimates; HbA1c, SII, and TSAT retained significance (empirical 95% CIs excluding 1.00). OR: odds ratio; CI: confidence interval; HbA1c: glycated haemoglobin; SII: systemic immune-inflammation index; TSAT: transferrin saturation.*

**Supplementary Table S4.** Non-invasive fibrosis indices (FIB-4 and APRI) across pattern groups.

| Group (n) | FIB-4, median (IQR) | FIB-4 low-risk, n (%) | APRI, median (IQR) |
| --- | --- | --- | --- |
| Normal (548) | 0.89 (0.61–1.28) | 478 (87.2%) | 0.19 (0.15–0.24) |
| Hepatocellular (105) | 0.84 (0.62–1.29) | 86 (81.9%) | 0.27 (0.22–0.34) |
| Cholestatic (77) | 0.80 (0.56–1.20) | 71 (92.2%) | 0.17 (0.13–0.19) |
| Overlap (29) | 0.89 (0.63–1.24) | 25 (86.2%) | 0.26 (0.20–0.33) |

*FIB-4 low-risk category defined as FIB-4 <1.3 (age <65 years) or <2.0 (age ≥65 years). No between-group difference for FIB-4 (Kruskal–Wallis p=0.72). All groups remained below the 0.5 APRI threshold for advanced fibrosis. FIB-4: fibrosis-4 index; APRI: AST-to-platelet ratio index.*

**Supplementary Figure S1. Study flow diagram according to STROBE reporting guidelines.**

**
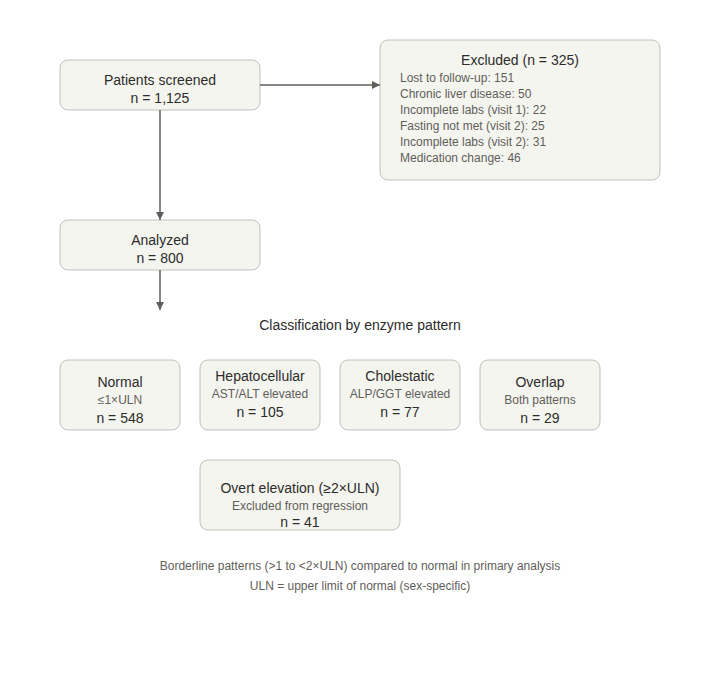
***Supplementary Figure S1. STROBE-style flow diagram of patient selection. Of 1,125 consecutively screened outpatients, 325 were excluded (reasons detailed in the figure), and 800 were analysed. Participants were classified by enzyme pattern using sex-specific upper limits of normal (ULN): normal (n=548), hepatocellular (n=105), cholestatic (n=77), overlap (n=29), and overt elevation (n=41). Borderline patterns (>1 to <2×ULN) were compared to the normal group in the primary analyses; the overt elevation group (≥2×ULN) was excluded from regression analyses. ULN: upper limit of normal.*

**Supplementary Figure S2. Graphical summary of pattern-based metabolic and inflammatory signatures in borderline liver enzyme elevation**.
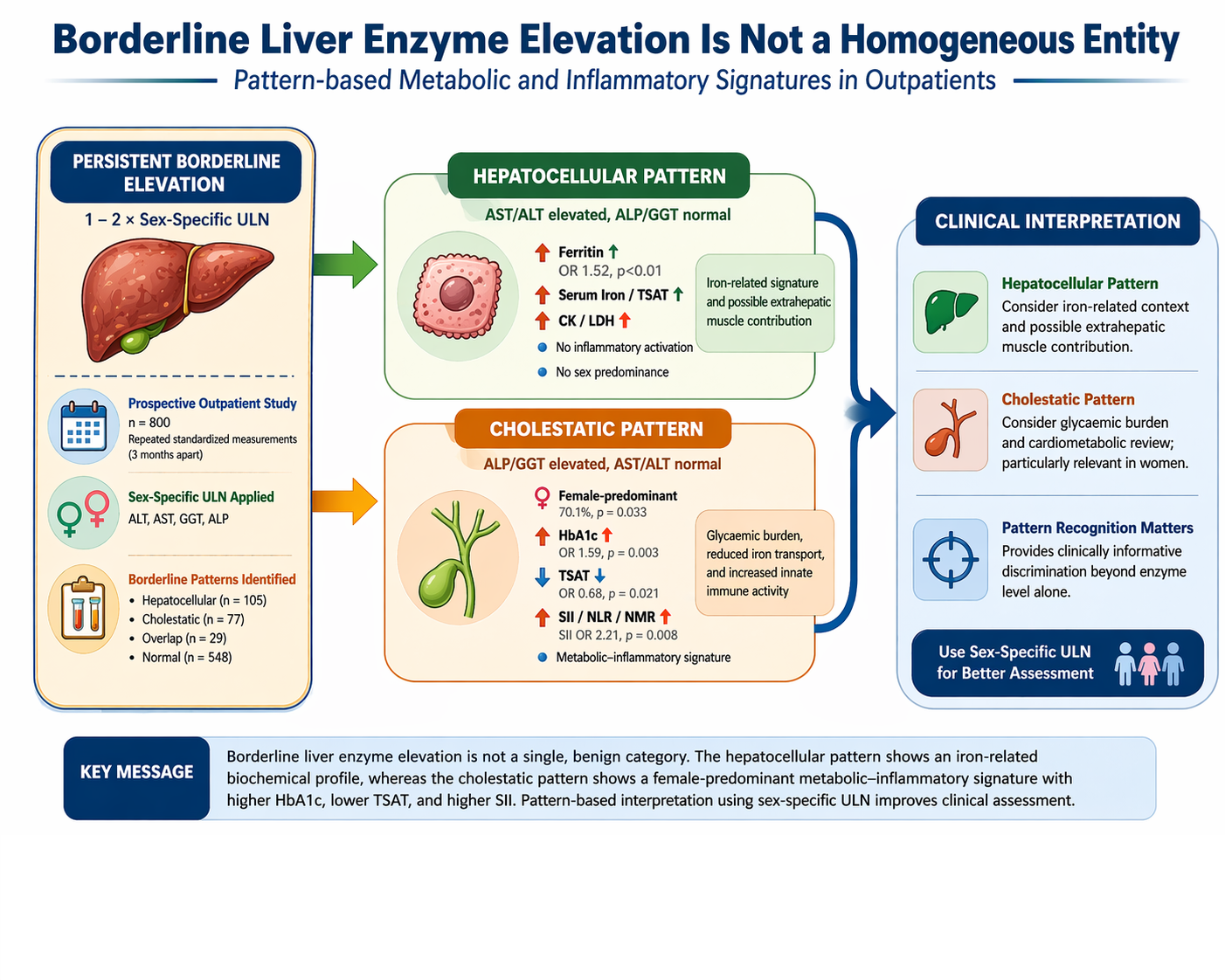
*Graphical abstract of the study. Among 800 outpatients evaluated for persistent borderline liver enzyme elevation (1–2× sex-specific ULN), three biochemically distinct subphenotypes were identified. The hepatocellular pattern (n=105) exhibited an iron-related biochemical signature — elevated ferritin (OR 1.52, p<0.01), serum iron/TSAT, and CK/LDH — without inflammatory activation or sex predominance. The cholestatic pattern (n=77) was female-predominant (70.1%, p=0.033) and characterised by a metabolic–inflammatory phenotype, with higher HbA1c (OR 1.59, p=0.003), lower TSAT (OR 0.68, p=0.021), and elevated systemic inflammatory indices (SII OR 2.21, p=0.008). An overlap pattern (n=29) combined features of both; 548 patients had normal enzyme levels. Pattern recognition using sex-specific upper limits of normal (ULN) provides clinically informative biochemical discrimination beyond the enzyme level alone. ULN: upper limit of normal; CK: creatine kinase; LDH: lactate dehydrogenase; TSAT: transferrin saturation; SII: systemic immune-inflammation index; NLR: neutrophil-to-lymphocyte ratio; NMR: neutrophil-to-monocyte ratio; OR: odds ratio.*
